# Comparison of Patterns of Non-suicidal Self-Injury and Emotion Dysregulation Across Mood Disorder Subtypes

**DOI:** 10.3389/fpsyt.2022.757933

**Published:** 2022-05-12

**Authors:** So Yung Yang, Dongbin Lee, Hyewon Jeong, Yunji Cho, Jae Eun Ahn, Kyung Sue Hong, Ji Hyun Baek

**Affiliations:** ^1^Department of Psychiatry, National Health Insurance Service Ilsan Hospital, Goyang-si, South Korea; ^2^Institute of Behavioral Sciences in Medicine, College of Medicine, Yonsei University, Seoul, South Korea; ^3^Department of Psychiatry, Samsung Medical Center, Sungkyunkwan University, Seoul, South Korea; ^4^Center for Clinical Research, Samsung Biomedical Research Institute (SBRI), Seoul, South Korea; ^5^School of Medicine, Sungkyunkwan University, Suwon, South Korea

**Keywords:** mood disorders, non-suicidal self-injury (NSSI), emotional dysregulation, bipolar II disorder (BD-II), childhood trauma

## Abstract

**Introduction:**

Non-suicidal self-injury (NSSI) is frequently encountered in patients with mood disorders. Emotion dysregulation (ED), frequently observed in mood disorders, could be a major mediating factor in NSSI. The aim of this study was to explore differences in NSSI behavior and ED across mood disorder subtypes. The relationships between childhood trauma and NSSI and ED were also explored.

**Methods:**

A total of 191 patients with mood disorders were included in this study. The patterns of NSSI behavior and ED across patients with bipolar I disorder (BD-I), bipolar II disorder (BD-II), and major depressive disorder (MDD) were compared.

**Results:**

More than half (54%) of the subjects experienced NSSI. Patients with BD-II and MDD engaged in NSSI behavior more frequently than those diagnosed with BD-I. NSSI behaviors in patients with BD-II most commonly included cutting, whereas hitting behaviors were most common among other groups. Patients with BD-II and MDD reported more severe ED than those with BD-I. In the case of childhood trauma, those with BD-II and MDD reported greater emotional neglect than those with BD-I. Structural equation modeling revealed that ED mediated the association between childhood trauma and NSSI.

**Conclusion:**

BD-I was associated with less frequent NSSI behavior and less severe ED than BD-II and MDD. ED mediated the association between childhood trauma and NSSI. Promoting emotion regulation strategies could prevent NSSI behavior in patients with mood disorders.

## Introduction

Non-suicidal self-injury (NSSI) has been defined as the deliberate and self-inflicted destruction of body tissues without suicidal intent (1). The prevalence of NSSI among the clinical samples of adults ranged from 13 to 37% (2–4). Typical NSSI behaviors include cutting, scraping skin, skin-picking, self-hitting and biting, burning, and tying (5, 6). NSSI usually begins in adolescence. It has the highest prevalence during adolescence and early adulthood, but it can manifest at any age (4, 7, 8). Although by definition, NSSI occurs without an intent to commit suicide, it is strongly associated with suicidal thoughts and behaviors (8, 9).

NSSI has received increasing attention over the past several decades (10) with growing evidence suggesting that it is a transdiagnostic symptom commonly associated with psychiatric disorders including mood disorders (11–13). While self-harm is a diagnostic criterion of borderline personality disorder, studies have shown that mood disorders and NSSI frequently co-exist (14, 15). Individuals who engaged in NSSI exhibited elevated levels of anxiety and depression compared to those who did not engage (16, 17). A meta-analysis showed that individuals with mood disorders exhibited more than twice the odds (odds ratio = 2.09) of engaging in NSSI compared to those without such disorders (18).

Emotion dysregulation (ED) is a factor that could mediate the relationship between mood disorders and NSSI behavior (19). ED refers to “an individual's ability to modify an emotional state so as to promote adaptive, goal-oriented behaviors” (20). Therefore, ED refers to the failure to change the reactivity of emotions or the unacceptance and devaluing of emotions (21). ED has been proposed as a critical component in the development and maintenance of mood disorders (19). NSSI is often used to regulate affect to reduce or escape from an aversive or negative affective state (22, 23). It may provide relief from emotional distress (24). Thus, NSSI is often intended to avoid the negative emotional experiences associated with mood disorders.

Previous studies demonstrated the association of mood disorders with NSSI and ED (13, 19). However, little is known about whether the patterns of NSSI behavior and ED differ by mood disorder subtypes such as bipolar disorder (BD) and major depressive disorder (MDD). In addition, differences in NSSI behavior and ED between patients with bipolar I disorder (BD-I) and bipolar II disorder (BD-II) remain unclear. Considering that BD-I, BD-II, and MDD differ clinically in terms of long-term illness trajectory (25–27), they might show different patterns of ED and NSSI behavior.

Childhood trauma is generally accepted as a risk factor for NSSI (28, 29) and mood disorders (30). Some studies have suggested that depression (31) and ED (24, 32) might play a potential role in the association between NSSI and childhood trauma. However, previous studies investigating the relationship between NSSI and childhood trauma mainly targeted adolescents (29). Few studies involved adult clinical samples.

The aim of this study was to determine differences in NSSI patterns and association with ED according to mood disorder subtypes (i.e., BD-I, BD-II, and MDD). The relationship between childhood trauma and NSSI and the possible mediating effects of ED on the relationship were also explored.

## Materials and Methods

### Study Participants

Study participants were recruited from the psychiatry outpatient clinic of Samsung Medical Center from January 2019 to November 2020. Subjects aged between 18 and 60 years who were diagnosed with BD-I, II, or recurrent MDD were included. Board-certified psychiatrists who had at least one year of research experience evaluated the participants' psychiatric diagnoses using DSM-V criteria. These study participants were clinically stable, i.e., they scored 3 (mildly ill) or lower on the Clinical Global Impression of Severity scale (33) at the time of assessment. Clinical severity was evaluated by the same psychiatrists who made the clinical diagnosis. Based on comprehensive psychiatric evaluations, we only included individuals who could reliably report their symptoms and past histories. All participants were undergoing standard pharmacological treatment, which included mood stabilizers or antidepressants. The other inclusion criterion was the absence of evidence of schizophrenia, organic mental disorder, intellectual disability, and substance or medical illness-induced mood disorders. Patients who could not reliably report their lifetime history were excluded. Information was collected using a checklist of demographic data, as well as psychiatric and medical history. The participants completed self-reported questionnaires related to childhood trauma, emotion regulation, and the lifetime frequency of NSSI. Written informed consent was obtained from all subjects after a complete explanation of the study. This study was approved by the Institutional Review Board (IRB) of Samsung Medical Center (IRB no. 2018-11-019).

### Measures

#### Non-suicidal Self-Injury

NSSI behaviors and functions were assessed using the Korean version of the Inventory of Statements about Self-Injury (ISAS) (5, 34). The first section of the ISAS measures the lifetime frequency of 12 NSSI behaviors including cutting, biting, carving, burning, pinching, pulling hair, severe scratching, banging or hitting self, interfering with wound healing, rubbing skin against a rough surface, sticking self with needles, and swallowing dangerous substances. In this section, the participants are also asked about the frequency of each behavior and the method they most commonly used.

#### Emotion Dysregulation

The Korean version of Difficulties in Emotion Regulation Scale (DERS) (35, 36) was used to measure ED. This 36-item scale asked the relevance of each item based on a 5-point scale. Difficulties in emotion regulation were assessed using six subscales: impulse control difficulties, lack of emotional awareness, non-acceptance of emotional response, lack of emotional clarity, limited access to emotion regulation strategies, and difficulties engaging in goal-directed behavior. The cumulative scores of the DERS subscales were also calculated.

#### Childhood Trauma

A childhood history of abuse was assessed retrospectively using the Korean version of the Child Trauma Questionnaire (CTQ) (37, 38). The CTQ is a 28-item self-reported assessment of the severity and frequency of childhood maltreatment, including physical, sexual, and emotional abuse, and physical and emotional neglect. The items were scored using a 5-point Likert scale ranging from 1 (never true) to 5 (very often true). The total score of the CTQ subscales was also calculated.

### Statistical Analysis

All statistical analyses were executed using IBM SPSS statistics version 23 (IBM Corp., Armonk, NY, USA) and SAS version 9.4 (SAS Institute Inc, Cary, NC, USA). The Shapiro-Wilk test was used to determine the normality of parametric variables. Variables that were normally distributed with equal variance among groups were compared using the Student's *t*-test or one-way ANOVA followed by Fisher's LSD *post-hoc* comparison. Data that were neither normally distributed nor had equal variance were tested using Mann-Whitney's U-test. Categorical variables were compared using the χ2-test.

The relationship between childhood trauma and NSSI and the mediating effect of ED on such relationships were explored via structural equation modeling (SEM) analysis using Markov Chain Monte Carlo (MCMC) with 1000 bootstrap samples in SPSS AMOS. Age and sex variables were included in the model as covariates to control for their potential confounding effects. Model fit was examined using the Comparative Fit Index (CFI), the Root Mean Square Error of Approximation (RMSEA), and the Standardized Root Mean Square Residual (SRMR). The acceptable fit of SEM was defined as CFI values above 0.90, RMSEA values <0.08, and SRMR values less than 0.08 (39, 40).

## Results

### Patterns of NSSI Behaviors and Emotion Dysregulation in Patients With Mood Disorders

No significant differences in sociodemographic variables including sex and age were found between the diagnostic groups (Table 1). Of all participants, 54% had a lifetime NSSI history (Table 2). When statistically analyzing the proportion of patients who experienced NSSI by diagnosis, patients with BD-I were less likely to engage in NSSI compared to other diagnostic groups (BD-I: 33.9%, BD-II: 64.4%, MDD: 58.1%, χ^2^ = 13.843, *p* = 0.001) (Table 2).

**Table 1:** Participants' sociodemographic characteristics.

	**Total subjects (*N* = 191)**	**1. BD-I (*N* = 56)**	**2. BD-II (*N* = 104)**	**3. MDD (*N* = 31)**	**F or χ^2^**	***p*-value**
Sex, male, n (%)	67 (35.1)	20 (35.7)	38 (36.5)	9 (29.0)	0.605^a^	0.739
Age, year, mean (SD)	30.3 (9.6)	32.1 (10.6)	29.5 (9.2)	29.7 (8.9)	1.495^b^	0.227
Education, high school graduate or more, n (%)	132 (69.5)	40 (71.4)	73 (70.2)	19 (63.3)	0.66^a^	0.719
Marital state, married (%)	56 (29.6)	22 (39.3)	24 (23.3)	10 (33.3)	4.68^a^	0.096
Occupation, present, n (%)	135 (71.4)	44 (78.6)	69 (67.0)	22 (73.3)	2.448^a^	0.294

*BD-I, bipolar I disorder; BD-II, bipolar II disorder; MDD, major depressive disorder; SD, standard deviation*.

a *Groups were compared using the one-way ANOVA test*.

b *Groups were compared using the χ2-test*.

**Table 2:** Descriptive statistics and differences in NSSI and DERS and CTQ scores between diagnostic groups.

	**Total subjects (*N* = 191)**	**1. BD-I (*N* = 56)**	**2. BD-II (*N* = 104)**	**3. MDD (*N* = 31)**	**F or χ^2^**	***p*-value^a^**	***Post-hoc* test^b^**
NSSI, present, *n* (%)	104 (54.5)	19 (33.9)	67 (64.4)	18 (58.1)	13.843	**0.001**	1 <2, 1 <3
DERS							
Total score, mean (SD)	107.9 (27.6)	97.2 (26.8)	112.2 (27.5)	113 (24.7)	6.326	**0.002**	1 <2, 1 <3
Impulse, mean (SD)	14.2 (5.8)	12.9 (5.4)	15 (6)	13.7 (5.6)	2.594	0.077	
Awareness, mean (SD)	19.8 (6.4)	18.8 (7)	20 (6.1)	20.9 (5.9)	1.257	0.287	
Acceptance, mean (SD)	22.8 (9)	20.3 (8.8)	23.6 (9.2)	24.8 (8)	3.337	**0.038**	1 <2, 1 <3
Clarity, mean (SD)	8 (3.3)	6.6 (3.2)	8.6 (3.3)	8.2 (2.9)	7.246	**0.001**	1 <2, 1 <3
Strategy, mean (SD)	19.1 (7.5)	16.4 (5.5)	20.1 (8.4)	20.5 (6.3)	5.306	**0.006**	1 <2, 1 <3
Goal, mean (SD)	13.9 (4.4)	12.3 (4.6)	14.7 (4)	14 (4.8)	5.494	**0.005**	1 <2
CTQ							
Total score, mean (SD)	54.5 (19.1)	49.2 (17.5)	56.2 (19.7)	58.4 (18.4)	3.252	**0.041**	1 <2, 1 <3
Emotional abuse, mean (SD)	12.2 (5.9)	10.8 (5.5)	12.8 (6.1)	13 (5.9)	2.37	0.096	
Physical abuse, mean (SD)	10.5 (5.6)	9.4 (5.9)	10.9 (5.4)	10.8 (5.4)	1.444	0.238	
Emotional neglect, mean (SD)	15.4 (6.2)	13.7 (6.5)	15.8 (6.1)	17 (5.2)	3.492	0.032	1 <2, 1 <3
Physical neglect, mean (SD)	9.5 (4)	8.8 (3.4)	9.7 (4.1)	10.5 (4.6)	2.034	0.134	
Sexual abuse, mean (SD)	6.9 (3.9)	6.6 (3.5)	7 (4.2)	7.1 (3.6)	0.295	0.745	

*BD-I, bipolar I disorder; BD-II, bipolar II disorder; MDD, major depressive disorder; CTQ, child trauma questionnaire; SD, standard deviation; NSSI, non-suicidal self-injury; DERS, difficulties in emotion regulation scale*.

a *Bold fonts indicate statistically significant differences with a p < 0.05*.

b *Fisher's LSD post-hoc comparisons (1, BD-I; 2, BD-II; 3, MDD)*.

**Figure 1:**
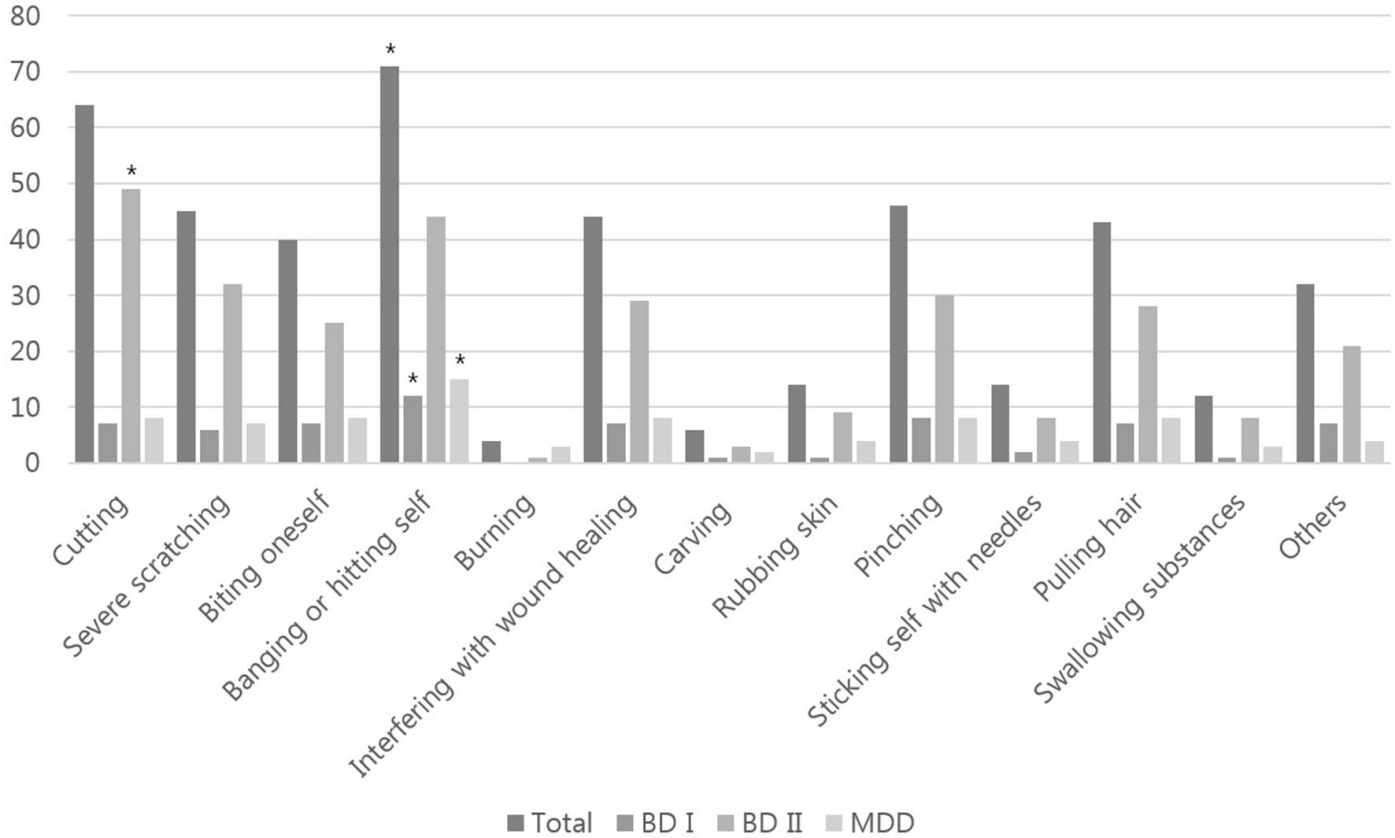
Types of NSSI behavior based on diagnostic group (multiple responses were allowed). BD-I, bipolar I disorder; BD-II, bipolar II disorder; MDD, major depressive disorder. Asterisk indicates the most commonly used self-injury method by the diagnostic group.

Figure 1 shows the different types of NSSI behavior (ISAS section 1) according to the diagnostic groups. When the methods ever used in their lifetime were queried (multiple responses allowed), “cutting” was the most common method, followed by “banging or hitting self” and “severe scratching” in patients with BD-II. In patients with BD-I or MDD, “banging or hitting self” was the most commonly used self-harm method, followed by “cutting,” “pinching,” and “severe scratching.” “Cutting” was the most commonly used method of self-harm, followed by “banging or hitting self,” “others,” and “interfering with wound healing” in patients with BD-II. However, “banging or hitting self” was the mainly used self-harm method in patients with BD-I and MDD (Supplementary Table 1).

Statistically significant differences between the diagnostic groups were detected in total DERS scores. The BD-I group reported lower DERS total scores, indicating less severe ED compared to the other diagnostic groups (F = 6.326, *p* = 0.002) (Table 2). This overall pattern was repeated for the DERS subscales except for impulse control difficulties and the lack of emotional awareness subscales. Patients with BD-I also reported a lower CTQ total score, indicating less severe overall childhood trauma experience compared to the other groups (CTQ total score: F = 3.252, *p* = 0.041). In sub-score analyses, the BD-I group scored significantly lower on the emotional neglect subscale than the other groups (F = 3.492, *p* = 0.032).

### Comparison Between Patients With and Without a Lifetime History of NSSI

Table 3 shows differences in the variables based on NSSI experience. A significantly greater proportion of females than males reported engaging in NSSI (χ^2^ = 5.189, *p* = 0.023). The NSSI group was younger (z = −3.659, *p* < 0.001) and more frequently unmarried (χ^2^ = 8.345, *p* = 0.004). Participants with and without NSSI did not differ in educational level or occupational status. As for the clinical variables, the NSSI group reported significantly higher levels of childhood traumatic experience (t = −6.145, *p* < 0.001) and difficulties in emotion regulation (t = −5.457, *p* < 0.001).

**Table 3:** Descriptive statistics and differences in variables between non-NSSI and NSSI groups.

	**NSSI (*N* = 104)**	**non-NSSI (*N* = 87)**	***t* or χ^2^**	***p*-value**
Sex, male (%)	29 (27.9)	38 (43.7)	5.189	0.023
Age, mean (SD)	27.63 (7.266)	33.46 (11.046)	4.215	<0.001
Education, high school graduate or more, *n* (%)	74 (71.2)	58 (67.4)	0.306	0.58
Marital state, married (%)	20 (19.4%)	33 (38.4%)	8.345	0.004
Occupation, present, *n* (%)	68 (66.0)	67 (77.9)	3.245	0.072
CTQ total score, mean (SD)	61.54 (18.90)	46.11 (15.79)	6.145	<0.001
DERS total score, mean (SD)	117.23 (24.44)	96.82 (27.23)	5.457	<0.001

*NSSI, non-suicidal self-injury; SD, standard deviation; CTQ, child trauma questionnaire; DERS, difficulties in emotion regulation scale*.

### Structural Equation Modeling Analysis

**Figure 2:**
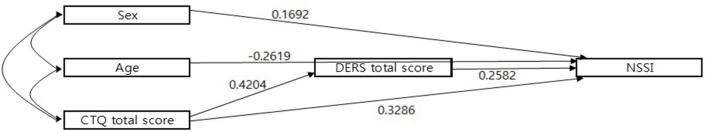
A conceptual model of the relationship between childhood trauma and emotion dysregulation (ED) and NSSI using standardized beta coefficients. Age and sex variables were included in the model as covariates. All paths were statistically significant. CTQ, child trauma questionnaire; DERS, Difficulties in Emotion regulation scale; NSSI, non-suicidal self-injury.

Table 4 presents the correlation of age with the total CTQ and DERS scores. Since age is known to be related to DERS scores, it was included in the correlation analysis. The rate of childhood trauma and severity of emotion dysregulation showed a significant positive correlation (r^2^ = 0.419, *p* < 0.001). Age was negatively correlated with ED (r^2^ = −0.229, *p* = 0.001) and childhood trauma (r^2^ = −0.177, *p* = 0.014). Figure 2 presents a conceptual model of the relationship between childhood trauma and ED and NSSI. Age and sex were included in the model as covariates to control for its potential confounding effect. The results showed that all paths were statistically significant. The model explained 36.4% of the NSSI of patients with mood disorders (total effect coefficient = 0.437). An increased incidence of childhood trauma directly predicted increased levels of NSSI (coefficient = 0.3286, 95% confidence interval (CI): 0.1629–0.4832). Childhood trauma was related to ED (coefficient = 0.4204, 95% CI: 0.2959–0.5334), which in turn, was significantly predictive of NSSI (coefficient = 0.2582, 95% CI: 0.0829–0.4256). Mediation analysis revealed that ED mediated the relationship between childhood trauma and NSSI (standardized indirect effect coefficient = 0.1086, 95% CI: 0.0332–0.1919; standardized total effect coefficient = 0.4372, 95% CI: 0.2857–0.5742). The model fit including all diagnostic groups was as follows: CFI = 0.943, RMSAEA = 0.103 (90% CI:0.029–0.183), SRMR = 0.045. The SEM results and fitness in each diagnostic group are presented in Supplementary Figure 1.

**Table 4:** Pearson product-moment correlation coefficients of variables.

	**1**	**2**	**3**
1. Age	-		
2. CTQ total score	−0.177^*^	-	
3. DERS total score	−0.229^**^	0.419^**^	-

* *P < 0.05*.

** *P < 0.01*.

## Discussion

NSSI is a widespread phenomenon without diagnostic boundaries. However, previous studies did not explore the differences across mood disorder subgroups. The current study investigated NSSI and ED patterns among patients with BD-I, BD-II, and MDD. We additionally analyzed the association of NSSI and ED with childhood trauma in patients with mood disorders.

Approximately 54% of the participants in our study reported a lifetime history of NSSI. The rate of NSSI in our study was comparable to that of a previous study (13). A previous study involving patients seeking treatment at a general practice clinic in the United States (U.S.) reported a higher prevalence of NSSI in patients with mood disorders than in those with other psychiatric disorders (43 vs. 20%, respectively) (13). The prevalence of NSSI was especially high in subjects with bipolar disorder (up to 52%) (13).

This was the first study that compared the rates and patterns of NSSI in patients with BD-I, BD-II, and MDD. Notably, patients with BD-I showed substantially lower rates of lifetime NSSI behavior than the other groups. The rate of lifetime NSSI behavior was the highest in the BD-II group. Previous studies evaluating the rate of NSSI behavior in patients with psychiatric diagnoses reported mixed results regarding NSSI rates in patients with BD (18, 41). The contrasting results of NSSI frequency between BD-I and BD-II patients might have contributed to the mixed results. The patterns of NSSI behavior also differed among the three groups. Patients diagnosed with BD-II manifested cutting as the most common method of NSSI, which is potentially associated with a high degree of tissue damage compared to the other methods.

Consistent with the higher rate of NSSI behavior in patients with BD-II and MDD, difficulties in ED were more severe in those groups. BD-II was associated with profound ED, similar to MDD, rather than BD-I. A meta-analysis revealed that ED was common in patients with BD, but the differences in ED between patients with BD-I and BD-II were not clear (42). A single study reported the absence of differences in DERS scores between patients with BD-I and BD-II (43). However, the small sample size affected the results. Previous studies reported significant ED in patients with mood disorders compared to the general population, which was pervasive across diverse mood states including manic, depressive, and euthymic conditions (44, 45). In particular, mood dysregulation is more severe in BD-II than in BD-I (46, 47), which corroborates our study findings. Decreased ED correlated with a decrease in depression and anxiety (42), suggesting an association between negative affect and persistent ED. Depressive episodes are known to be more frequent in patients with BD-II than BD-I (27). The more frequent depressive episodes and mood swings observed in patients with BD-II often resemble severe ED found in borderline personality disorder (48, 49). A neuroimaging study also suggested differences in mood regulation circuitry between patients with BD-I and BD-II (50).

Consistent with the significant differences in total DERS scores, patients with BD-I showed better emotion regulation than those manifesting BD-II and MDD on all subscales of the DERS, and the difference reached a significant level in four domains (non-acceptance of emotion response, lack of emotional clarity, limited access to emotion regulation strategies, and difficulties engaging in goal-directed behavior). In contrast to our study, no previous studies analyzed the differences in DERS subscales between mood disorder subtypes. In a previous meta-analysis, subjects with BD showed significantly higher DERS subscale scores except for the awareness subscale score compared to healthy controls (42). Compared to borderline personality disorder, BD was associated with significantly lower scores on all the DERS subscales. A previous study investigating the latency profiles of DERS reported that the awareness subscale did not correlate with other subscale scores (51). Understanding the ED profiles across mood disorder subtypes will facilitate treatment strategies for these populations. Further studies are needed to confirm the study findings.

Although NSSI implies non-suicidal intent, it is associated with an increased risk of suicidal attempts (8, 9). The higher the rate of NSSI behavior, the more frequent the use of methods with the potential for a high degree of tissue damage, and greater ED in patients with BD-II might contribute to the increased suicide risk in patients with BD-II. A recent clinical study with the largest-ever sample size (52) and a meta-analysis (53) confirmed the higher prevalence of suicide attempts in patients with BD-II. In a recent prospective study of patients diagnosed with BD and MDD (54, 55), suicide attempts were more frequently observed in patients with BD than in those with MDD mainly because of the higher duration of high-risk illness, i.e., more frequent depressive episodes. Subjects in the MDD group in our study showed NSSI rates comparable to those in the BD-II group, reinforcing the association between NSSI behavior and recurrent depression.

In our study, the CTQ scores were also higher in patients with BD-II and MDD than in patients with BD-I. However, in the sub-scale analyses, such differences were significant only for the emotional neglect subscale. Emotional neglect is arguably the most subjective and difficult to define among the forms of abuse (56). A prior study reported no differences in the rate of childhood traumatic experience of patients with BD-I and BD-II (57). Further studies are needed to confirm our findings.

In accordance with previous studies (8, 58), the NSSI group was younger and included proportionately higher numbers of females than the non-NSSI group. The CTQ and DERS scores differed depending upon the diagnosis, with NSSI being more prevalent in the group with higher CTQ and DERS scores. These results support the mediating role of ED on the association between childhood trauma and NSSI. As hypothesized, the SEM results revealed that ED mediated the relationship between childhood trauma and NSSI. When evaluating the model fit using total subjects including all diagnostic groups, a single indicator was not acceptable (RMSAEA), probably due to heterogeneity between the diagnostic groups. Subgroup analyses showed a better fit except for the BD-II group for unknown reasons. Diagnostic characteristics may also affect the model fit. The correlation between CT and NSSI has been confirmed in studies enrolling various subjects as well as clinical samples (29). A single study explored the mediating role of ED in the relationship between CT and NSSI, although it involved adolescent inpatients (59). A study of adult clinical samples has yet to be reported. The current study further emphasized the importance of ED as a mediating factor by limiting the patient group to those with mood disorders. ED is a major independent risk factor for NSSI (21, 60). The functional aspects of ED in NSSI have been studied, especially in terms of behavioral theory. Based on the behavioral model, the positive reinforcement function (e.g., to feel something) and negative reinforcement function (e.g., to relieve depression or uncomfortable internal experiences) of NSSI might be relevant to individuals with ED (22, 61). In this respect, individuals who experience negative emotions may use NSSI as a coping strategy. A group skills training program in dialectical behavior therapy is one possible treatment option that appears to be effective in decreasing emotional reactivity and improving psychological wellbeing in patients with bipolar disorder (62). A further study is needed to identify better strategies to decrease ED and NSSI in patients with mood disorders.

This study had several limitations. First, all measures were evaluated using self-reported questionnaires. In particular, the retrospective study may be associated with recall bias. However, a previous study showed that recall bias accounted for <1% of the reported variance in measures of childhood abuse (63). Second, since the data included patients with mood disorders only, it was not clear if the current study results apply only to mood disorders or the findings could be generalized to other clinical samples. Third, this study was performed as a cross-sectional and single-center study of patients who were treated in a single hospital. Therefore, prospective and multicenter studies are needed before the results can be generalized. Fourth, SEM showed that childhood trauma and ED only explained part of the mechanism of NSSI. Further studies that include diverse factors that contribute to the development of NSSI are needed.

## Conclusions

In conclusion, NSSI is common across diverse mood disorders subtypes. Patients with BD-I had the lowest prevalence of NSSI and significantly less ED compared to those diagnosed with BD-II and MDD. The association between NSSI and childhood trauma was also mediated by ED. Thus, it could be beneficial to promote emotional coping skills in patients with mood disorders and a history of childhood trauma to prevent NSSI.

## Data Availability Statement

The raw data supporting the conclusions of this article will be made available by the authors, without undue reservation.

## Ethics Statement

The studies involving human participants were reviewed and approved by Institutional Review Board (IRB) of Samsung Medical Center. The patients/participants provided their written informed consent to participate in this study.

## Author Contributions

JB designed the study and wrote the protocol. JB and KH obtained the funding. DL, YC, and JA collected and managed the data. SY managed the literature searches and analyses. SY, DL, and HJ performed the statistical analysis, and SY and JB wrote the first draft of the manuscript. All authors contributed to the final manuscript and have approved it.

## Funding

This work was supported by the Korea Medical Device Development Fund grant funded by the Korean government (the Ministry of Science and ICT, the Ministry of Trade, Industry and Energy, the Ministry of Health & Welfare, and the Ministry of Food and Drug Safety) [Project Numbers NTIS 9991006915 and KMDF_PR_20200901_0250] and the Original Technology Research Program for Brain Science through the National Research Foundation of Korea (NRF) funded by the Ministry of Science and ICT [No. 2019M3C7A1030624].

## Conflict of Interest

The authors declare that the research was conducted in the absence of any commercial or financial relationships that could be construed as a potential conflict of interest.

## Publisher's Note

All claims expressed in this article are solely those of the authors and do not necessarily represent those of their affiliated organizations, or those of the publisher, the editors and the reviewers. Any product that may be evaluated in this article, or claim that may be made by its manufacturer, is not guaranteed or endorsed by the publisher.

## Supplementary Material

Supplementary Figure 1 A conceptual model of the relationship between childhood trauma and emotion dysregulation and NSSI in each diagnostic group. Age and sex were included in the model as covariates. Statistically significant paths are indicated with an asterisk (^*^). The BD-I and MDD models showed acceptable fit (CFI = 1.000, RMSAEA = 0.000 (90% CI: 0.000–0.169), with SRMR = 0.024 and CFI = 1.000, RMSAEA 0.000 (90% CI: 0.000–0.329) and SRMR = 0.053, respectively). The BD-II model showed poor fit (CFI = 0.850, RMSAEA = 0.213 (90% CI: 0.105–0.340), SRMR = 0.079). CTQ, child trauma questionnaire; DERS, difficulties in emotion regulation scale; NSSI, non-suicidal self-injury; BD-I, bipolar I disorder; BD-II, bipolar II disorder; MDD, major depressive disorder; CFI, comparative fit index; RMSEA, root-mean-square error of approximation; SRMR, standardized root-mean-square residual.

Supplementary Table 1 Main form of individual's NSSI behaviors by diagnotic groups.
