# Supplementary figures and images for: Comparison of Patterns of Non-suicidal Self-Injury and Emotion Dysregulation Across Mood Disorder Subtypes
Source: Front Psychiatry. 2022 May 12;13:757933. doi: 10.3389/fpsyt.2022.757933 (PMC9133457; doi:10.3389/fpsyt.2022.757933)

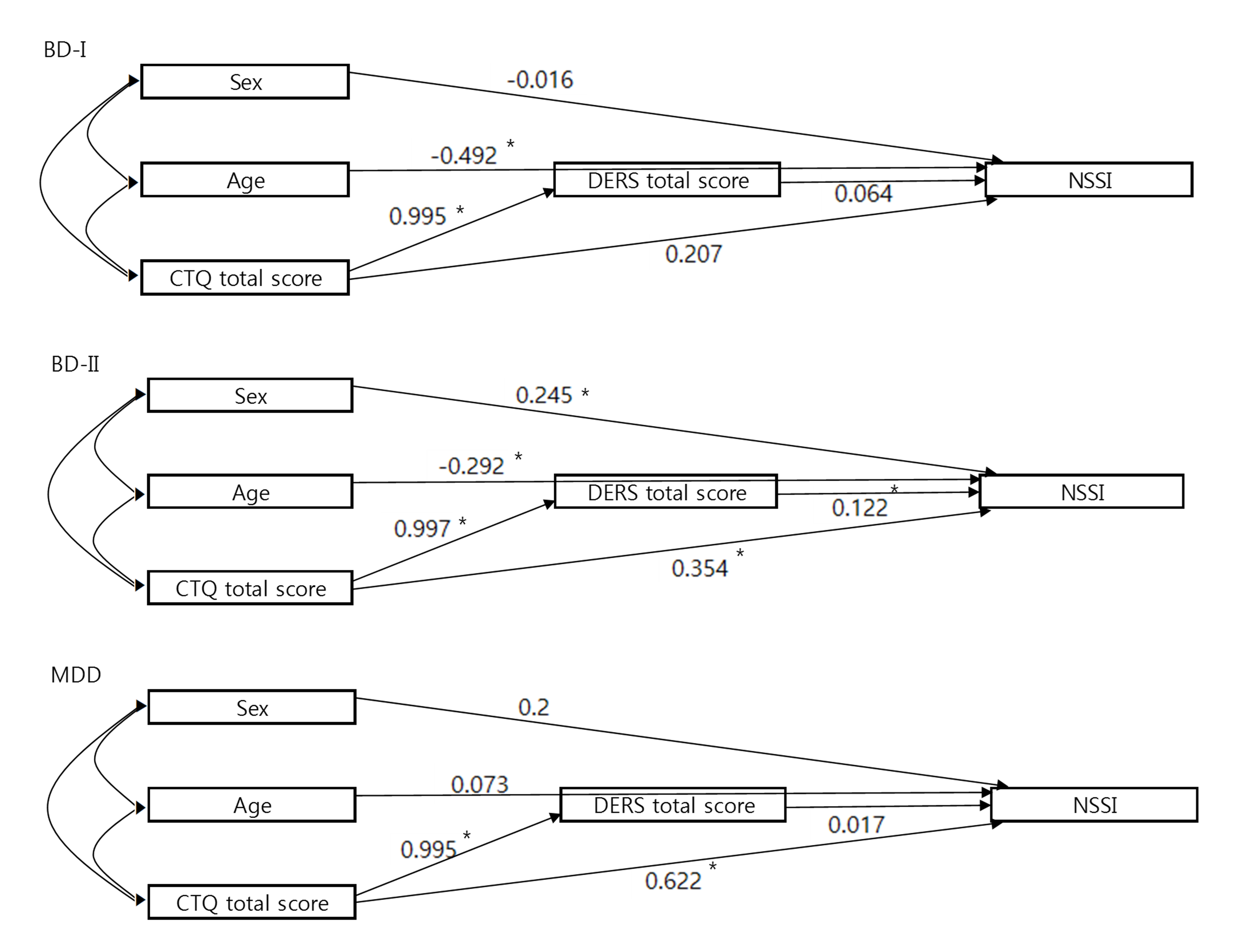

Supplement: Supplementary Figure 1 — A conceptual model of the relationship between childhood trauma and emotion dysregulation and NSSI in each diagnostic group. Age and sex were included in the model as covariates. Statistically significant paths are indicated with an asterisk (*). The BD-I and MDD models showed acceptable fit (CFI = 1.000, RMSAEA = 0.000 (90% CI: 0.000–0.169), with SRMR = 0.024 and CFI = 1.000, RMSAEA 0.000 (90% CI: 0.000–0.329) and SRMR = 0.053, respectively). The BD-II model showed poor fit (CFI = 0.850, RMSAEA = 0.213 (90% CI: 0.105–0.340), SRMR = 0.079). CTQ, child trauma questionnaire; DERS, difficulties in emotion regulation scale; NSSI, non-suicidal self-injury; BD-I, bipolar I disorder; BD-II, bipolar II disorder; MDD, major depressive disorder; CFI, comparative fit index; RMSEA, root-mean-square error of approximation; SRMR, standardized root-mean-square residual. [file Image_1.TIF]
